# Models and detection of spontaneous recurrent seizures in laboratory rodents

**DOI:** 10.24272/j.issn.2095-8137.2017.042

**Published:** 2017-07-18

**Authors:** Bin Gu, Katherin A. Daltone

**Affiliations:** ^1^Department of Cell Biology and Physiology, University of North Carolina, Chapel Hill, NC 27599, USA;; ^2^Psychology & Neuroscience Program, University of North Carolina, Chapel Hill, NC 27599, USA

**Keywords:** Spontaneous recurrent seizures, Animal model, Epilepsy

## Abstract

Epilepsy, characterized by spontaneous recurrent seizures (SRS), is a serious and common neurological disorder afflicting an estimated 1% of the population worldwide. Animal experiments, especially those utilizing small laboratory rodents, remain essential to understanding the fundamental mechanisms underlying epilepsy and to prevent, diagnose, and treat this disease. While much attention has been focused on epileptogenesis in animal models of epilepsy, there is little discussion on SRS, the hallmark of epilepsy. This is in part due to the technical difficulties of rigorous SRS detection. In this review, we comprehensively summarize both genetic and acquired models of SRS and discuss the methodology used to monitor and detect SRS in mice and rats.

## INTRODUCTION

Epilepsy, a chronic neurological disorder that is characterized by spontaneous recurrent seizures (SRS), is the fourth most common neurological disorder (Hirtz et al., 2007). Epilepsy was first described over 2 500 years ago, yet there is still relatively little known about the underlying cause and currently no disease-modifying therapies exist. Current treatment options include antiepileptic drugs (AEDs), ketogenic diet, neurosurgical resection, and electrical stimulation of the central nervous system (CNS), which work for some but not all afflicted individuals (Laxer et al., 2014). Thus, there is an urgent unmet clinical need to discover treatments for the entire epileptic population. Most currently available AEDs were first identified using simple acute seizure models (i.e., pentylenetetrazol induced seizure and maximal electroshock seizure models) (Löscher, 2011). These acute models fail to mirror the spontaneous nature of seizures seen in epilepsy. This issue is hypothesized to contribute to the large percentage of epileptic patients (∼30%) for whom AEDs fail to prevent or control SRS. Therefore, studying epilepsy using laboratory animals exhibiting SRS will provide an important tool to explore the underlying mechanism of epilepsy and develop novel therapeutic approaches. 

Epilepsy has been studied in a wide range of species of laboratory animals from simple organisms (e.g., *Drosophila melanogaster*, *Caenorhabditis* *elegans* and *Danio rerio*) to non-human primates. Along this spectrum, *Rattus norvegicus* (rat) and *Mus musculus* (mouse) are the two most commonly used laboratory animals given their small size, docility, rapid breeding, and availability of advanced genetic tools. Importantly, rat and mouse models provide good construct, face, and predictive validities of epilepsy and demand relatively low cost and maintenance for chronic study of SRS. In this review, we discuss the methodology of SRS recording, and summarize both genetic and acquired models of SRS in rat and mouse, with particular emphasis on modeling and detection of SRS. Mechanism and treatment of epileptogenesis are addressed in other reviews (Goldberg & Coulter, 2013; Löscher et al., 2013; McNamara et al., 2006; Pitkänen & Lukasiuk, 2011; Varvel et al., 2015).

## MONITORING AND DETECTION OF SRS IN RODENTS

**Figure 1:**
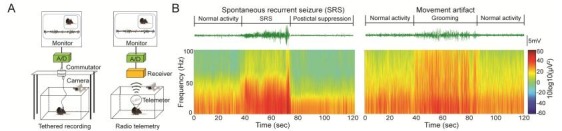
Schematic of video-EEG recording and EEG analyses

Chronic recording and detection of SRS in rodents is fundamental for preclinical research of epilepsy. Rigorous monitoring of SRS requires continuous time-locked video-EEG 24/7 in freely moving rodents. To capture biopotentials of the brain, most studies utilize single or multiple unipolar or bipolar recording electrodes which are intracranially placed. Skull or intracerebral electrode arrays are also used to cover broader brain regions. EEGs are acquired via either tethered or telemetry (wireless) recording systems in free-roaming, conscious rodents (Figure 1A). If a telemeter is used, it is either directly mounted on the head or tunneled and secured subcutaneously on the back or abdomen of rodents, providing the advantage of eliminating a wired interface between the animal and instrumentation. This minimizes the electrical noise and movement artifacts inherent in a tethered system. An inductive charging technique enables the telemeter to work 24/7 without the interruption of recharging the batteries. 

Given the rare, unpredictable nature and extremely diverse morphologies of SRS, identification of SRS is a technically challenging task. In most basic research settings, off-line visual inspection of EEG is performed by investigators to identify possible discrete epileptiform episodes, which are further confirmed by reviewing the time-locked video for behavioral correlates. Typical electrographic SRS features rhythmic neuronal firing characterized by increase of frequency and amplitude (especially in the gamma band) with clear initiation, propagation and termination (Figure 1B, left panel). In rodents, discrete epileptic discharges typically last seconds and are frequently followed by postictal suppression, which lasts minutes until normal electrographic activities resume. Electrographic SRS coincide with behavior phenotypes including rigid posture, facial automatisms, myoclonus, jumping and wild running, loss of postural control, tonic hindlimb extension, and death, which can be further semi-quantified using modified Racine’s scale (Ben-Ari, 1985; Racine, 1972). Spontaneous absence seizures characterized by spike-wave discharges (SWD) and behavioral arrest are also frequently observed in some models. 

To achieve successful SRS monitoring and detection, the following factors also need to be considered: (1) depending on models, SRS are relatively rare and tend to cluster. The seizure-free latent or interictal period may last days or even weeks before first or subsequent SRS emerge. Therefore, long-term (weeks to months) recording is required to achieve meaningful interpretation; (2) in most studies, brain areas covered by electrodes are limited. Electrographic seizures may occur out of the recording site, and in the absence of overt behavior change; (3) rodents are commonly singly housed during monitoring to minimize damage of recording device and facilitate video analysis. How social isolation affects SRS needs to be evaluated; (4) to visualize animal behavior during dark cycles, in some studies, the recording area is illuminated, thereby disrupting the normal light/dark cycle of monitored animals. Infrared light and imaging devices are recommended for behavior monitoring during dark cycle if circadian rhythm is considered (Cho, 2012; Hofstra & De Weerd, 2009); (5) SRS automatic detection algorithm is available, but manual validation is strongly recommended.

## SRS IN RODENT MODELS OF EPILEPSY

### SRS in genetic models of epilepsy

Approximately 40% of epilepsies are idiopathic. Genetics play a significant role in the development, maintenance, and difficulty of treating epilepsy. A growing number of epilepsy-related single gene mutations have been identified. Animals possessing analogous genetic manipulations (engineered or spontaneous) have proven useful in the search for the possible treatment for idiopathic epilepsy (Table 1). 

**1:** SRS in transgenic models of epilepsy

Gene	Modification	Latency	Frequency and features of SRS	References
*Scn1a*	*Scn1a*^-/-^	P9	Generalized convulsive SRS. SUDEP at P15	Yu et al., 2006
	*Scn1a*^+/-^	P21–P27	EEG and/or behavioral SRS lasted 20 s. Sporadic SUDEP from P21^*^	
	*Scn1a*^Flox/+^*::* *Zp3-Cre^+/-^*	N/A	12 out of 23 mice exhibited behavioral SRS (3 times/day, lasted 35 s). Lifespan of P33	Dutton et al., 2013
	*Scn1a*^Flox/+^*::* *Ppp1r2-Cre^+/-^*	N/A	2 out of 6 mice exhibited behavioral and/or EEG SRS	
	*Scn1a*^R1407X/R1407X^	P12–P16	Multiple tonic-clonic SRS/day confirmed by EEG (lasted 1–3 min, interval: 1–4 hr). SUDEP by P21	Ogiwara et al., 2007
	*Scn1a*^R1407X/+^	P18	Sporadic SUDEP in 1–3 mo^*^	
	*Scn1a*^R1648H/R1648H^	P16	Behavioral SRS lasted 30–90 s. SUDEP P16–P26	Martin et al., 2010
	*Scn1a*^R1648H/+^	N/A	2 out of 14 mice exhibited 21 SRS in total during 96 h EEG recording	
*Scn2a*	*Scn2a*^Q54^	2 mo^*^	EEG and behavioral SRS. Frequency and duration of SRS increased with age^*^	Kearney et al., 2001
*Scn8a*	*Scn8a*^8J/+^*, Scn8a*^med/+^* or Scn8a*^med-jo/+^	N/A	SWD with behavioral arrest^*^	Papale et al., 2009
	*Scn8*^N1768D/N1768D^	3 wk	No SRS prior to day of SUDEP 3 wk. SRS lasted <1 min	Wagnon et al., 2015
	*Scn8*^N1768D/+^	8–16 wk	0–3 SRS/day. SRS lasted <1 min. SUDEP 14 wk	
	*Scn8*^N1768D/-^	8 wk	As many as 25 SRS/day. SUDEP 9 wk	
*Scn1b*	*Scn1b^-/-^* or *Scn1b^del/del^*	P10	EEG and behavioral SRS at random intervals with duration from seconds to minutes. SUDEP 3 wk	Chen et al., 2004; 2007
*Kcnq2*	*Kcnq2*^A306T/A306T^	P24	Generalized EEG and behavioral SRS. SUDEP P16–P32^*^	Singh et al., 2008
*Kcnq3*	*Kcnq3*^G311V/G311V^	2 wk	Generalized EEG and behavioral SRS. SUDEP P0–P73^*^	Singh et al., 2008
*Kcna1*	*Kcna1*^-/-^	3 wk	Behavioral SRS lasted 20 s–2 min once or twice/hr throughout adult life. SUDEP 3–5 wk	Smart et al., 1998
*Kcna2*	*Kcna2*^-/-^	N/A	Tonic-clonic SRS. SUDEP at P17	Brew et al., 2007; Douglas et al., 2007
*Kcnmb4*	*Kcnmb4^-/-^*	N/A	Generalized EEG seizures without overt behavioral manifestation	Brenner et al., 2005
*Cacna1a*	Deletion (α_1A_^−/−^)	N/A	Absence seizures. SUDEP 3–4 wk	Jun et al., 1999
*Gria2*	*Gria2*^+/ΔECS^	P12	Behavioral SRS (once/4 hr). SUDEP by P20	Brusa et al., 1995
*Chrna4*	*Chrna4*^S252F/S252F or +^ and *Chrna4*^+L264/+L264 or +^	N/A	SRS with high-amplitude, low-frequency cortical EEG activity, prominent theta and delta waves	Klaassen et al., 2006
*Gabrg2*	*Gabrg2*^+/-^ or *Gabrg2*^+/R43Q^	P20	Behavioral arrest and associated SWD^*^ (up to 50 times/hr and variable)	Reid et al., 2013; Tan et al., 2007
*Tsc1/2*	*Tsc1/2^flox/flox^::GFAP-Cre*	2–3 wk	Generalized tonic-clonic SRS. Few SRS at 3 wk, frequency increased over time. SUDEP 7–10 wk	Zeng et al., 2011
*Fgf13*	*Fgf13*^+/-^	P15	Behavioral and EEG SRS. Frequency and duration varied by animal	Puranam et al., 2015
*Lgi1*	*Lgi1*^-/-^	P10	Clonic SRS (1.6 seizures/hr at P14). SUDEP at P20	Chabrol et al., 2010
*BACE1*	*BACE1*^-/-^	N/A	<40% rats exhibited generalized SRS and/or absence seizures	Hitt et al., 2010
*APP*	*APdE9*	N/A	65% exhibited SRS, 10%–15% mortality at any age but peak around 3–4 mo	Minkeviciene et al., 2009
	*hAPP_FAD_*	N/A	Spontaneous nonconvulsive seizure activity. Occurrence of SUDEP	Palop et al., 2007
*Ube3a*	*Ube3a*^m+/p-^	P18	SWD accompanied by behavioral immobility or tonic-clonic SRS^*^	Miura et al., 2002, Jiang et al., 1998a; 2010
*Mecp2*	*Viaat-Mecp2^−/y^* *Mecp2^308/y^*	5 wk N/A	Spontaneous rhythmic EEG activity including SWD^*^ Spontaneous behavioral myoclonic jerks	Chao et al., 2010 D'Cruz et al., 2010
*Shank3*	*Shank3* *OE*	N/A	Hyperexcitability discharges accompanied by EEG SRS	Han et al., 2013
*CNTNAP2*	*CNTNAP2^-/-^*	6 mo	SRS with generalized interictal spike discharges	Peñagarikano et al., 2011
*Epm2A*	*Epm2A^-/-^*	<9 mo	80% exhibited myoclonic SRS, more frequent during dark cycle	Ganesh et al., 2002
*Celf4*	*Celf4^Ff/Ff^ or Celf4^Ff/+^*	3 mo	Recurrent tonic-clonic seizures or absence seizures^*^	Yang et al., 2007
*Map2k1*	*caMEK1^flox/flox^::Nestin-Cre*	6–8 wk	Lifetime behavioral arrest and forelimb myoclonus (6.2 SRS/7 hr)	Nateri et al., 2007
^*^: model or strain dependent phenotype; ECS: editing site complementary sequence; OE: overexpression; SRS: spontaneous recurrent seizures; SUDEP: sudden unexpected death in epilepsy; SWD: spike-wave discharges.				

### Ion channel genes

Ion channels control the electrical transduction of cells, thereby playing a pivotal role in regulating neuronal excitability. Most epilepsy-related genes encode proteins composing voltage- or ligand-gated ion channels. Below we summarize genetic models of epilepsy that result from mutations in various types of ion channels.

Of the many ion channels, a number of disruptions in genes encoding voltage-gated sodium channels have been described in multiple human epilepsies, including genetic epilepsy with febrile seizures plus (GEFS+) and Dravet syndrome. Disruptions of genes encoding either α (SCN1A, SCN2A and SCN8A) or β (SCN1B) subunits of voltage-gated sodium channels are sufficient to trigger SRS in rodents (Chen et al., 2004, 2007; Dutton et al., 2013; Kearney et al., 2001; Martin et al., 2010; Ogiwara et al., 2007; Papale et al., 2009; Wagnon et al., 2015; Yu et al., 2006). In addition, two modifier loci (*Moe1* and *Moe2*) and multiple candidate modifier genes that influence the *Scn2a^Q54^* epilepsy phenotype have also been identified and refined (Hawkins & Kearney, 2012). 

Potassium channels also play an important role in action potentials by helping to return the neuron back to its resting membrane potential. *Kcna1* and *Kcna2* encode a pair of proteins (Kv1.1 and 1.2) which are members of the voltage-dependent potassium channel subfamily A. *Kcna1 *or *Kcna2* knockout mice display frequent, severe SRS throughout their lives. In addition, SRS caused death in 50% of *Kcna1* or *Kcna2* knockout mice beginning from three weeks of age (Brew et al., 2007; Douglas et al., 2007; Smart et al., 1998). Mutations of *Kcnq2* and *Kcnq3*, which encode subfamily Q of voltage-gated potassium channels have been found in patients with benign familial neonatal convulsions (BFNC). *Kcnq2* or *Kcnq3* mutant mice exhibit early onset generalized tonic-clonic SRS concurrent with M-current defects (Singh et al., 2008). Mice carrying *Scn2a^Q54^* transgene together with *Kcnq2* mutations (*Szt1* or V182M) result in an exacerbated epileptic phenotype (Kearney et al., 2006). A gain-of-function mutation of gene *Kcnmb4,* which encodes calcium-activated potassium channel accessory β4 subunit also led to SRS (Brenner et al., 2005). 

Calcium channels are important for neuronal excitability and intracellular signaling. Activation of T-type calcium channels evoke burst-firing in the thalamocortical circuitry that gives rise to SWD associated with absence epilepsy (Chen et al., 2014; Cheong & Shin, 2013). α1G T-type calcium currents play a critical role in the genesis of spontaneous absence seizures resulting from hypofunctioning P/Q-type channels (*α1_A_^−/−^*) (Jun et al., 1999; Song et al., 2004). These attacks have also been shown to reflect absence seizures in *tottering* (*tg*), *leaner* (*tg^la^*) and *rocker* (*rkr*) mice, which have spontaneously occurring mutant (Fletcher et al., 1996; Jun et al., 1999; Zwingman et al., 2001). In addition to pore-forming α1 subunit, loss of function mutations in ancillary subunits of calcium channels, including naturally occurring mutations in the β subunit gene *Cchb4* in the lethargic (*lh*) mouse, loss of α2δ2 subunit protein in *ducky* mouse (*du* and *du^2j^*) and dysfunctional calcium channel γ2 subunits in *stargazer* (*stg*) and *waggler* (*wgl*) mice also result in SRS (Burgess et al., 1997; Zamponi et al., 2010).

In addition to voltage-gated ion channels, mutations of ligand-gated ion channel genes also result in SRS in mice. Heterozygous mice carrying an editing-deficient GRIA2 subunit allele express AMPA receptors with increased calcium permeability and develop SRS (Brusa et al., 1995). Fast ionotropic nicotinic acetylcholine receptor (nAChR) subunit genes, α2 (*Chrna2*), α4 (*Chrna4*) and β2 (*Chrnb2*), have been affiliated with autosomal dominant nocturnal frontal lobe epilepsy (ADNFLE) when mutated. Mice with *Chrna4* mutations (*Chrna4*^S252F^ or *Chrna4*^+L264^) exhibited frequent SRS with diverse seizure semiology ranging from behavioral arrest to convulsive jerking (Klaassen et al., 2006). GABA_A_ γ2-subunites have five known seizure associated mutations. Of these mutations, the R43Q mutation is of particular interest because it is related to childhood absence epilepsy and febrile seizures (Wallace et al., 2001). Both heterozygous *Gabrg2* knock-out and R43Q knock-in mice exhibited spontaneous absence seizures accompanied by SWD (Reid et al., 2013; Tan et al., 2007). 

### Non-ion channel genes

SRS are also related to interruptions of non-ion channel genes that are involved in diverse neurological disorders including tuberous sclerosis complex (TSC), Alzheimer’s disease (AD) and autism. Notably, SRS can arise as a comorbid phenotype and/ or secondary consequence of gene modification from germline. 

Epilepsy is the most common presenting symptom in TSC. Up to 80%–90% of individuals with TSC will develop epilepsy during their lifetime. Two genes, *TSC1* and *TSC2*, encoding the proteins hamartin and tuberin, respectively, have been identified as causing TSC. Both genes, when conditionally inactivated in mice, have been shown to contribute to epileptic phenotype, among which *Tsc2* led to more severe and frequent seizures (Zeng et al., 2011).

Prevalence of epilepsy in Alzheimer’s disease is significantly higher than in age-matched control populations. Manipulation of AD related genes (e.g., *BACE1* and *APP*) can also cause SRS in mice. One study showed that *BACE1* knockout mice were predisposed to both spontaneous and chemically induced seizures (Hitt et al., 2010). Autosomal-dominant mutations in amyloid precursor protein (APP) cause hereditary early-onset familial Alzheimer's disease (FAD). Transgenic mice overexpressing a mutant form of human APP (*hAPP_FAD_*) have spontaneous nonconvulsive seizure activity in cortical and hippocampal networks (Palop et al., 2007). It was shown that 65% of mice carrying human APP with Swedish double mutation (*APPswe*) cointegrated with human preselinin-1 with exon 9 deletion (*PS1dE9*) exhibited unprovoked seizures (Minkeviciene et al., 2009; Um et al., 2012). 

Autism spectrum disorder (ASD) related genes are also extensively studied given the fact that epilepsy is common in individuals with autistic-like behavior resulting from particular genetic predisposition. A null mutation of maternal *Ube3a* gene (exon 1–2 or exon 15 and 16) results in core pathologies of Angelman syndrome including spontaneous EEG abnormality in mice (Jiang et al., 1998b; Miura et al., 2002). Spontaneous behavioral seizures were witnessed in mice with 1.6Mb large deletion (*Ube3a* to *Babrb3*) and loss of *Ube3a* selectively from the GABAergic neurons (Jiang et al., 2010; Judson et al., 2016). Global or conditional manipulation of *Mecp2* gene in Rett syndrome model mice is also sufficient to elicit SRS, including spontaneous epileptiform discharges (Chao et al., 2010; D'Cruz et al., 2010; Shahbazian et al., 2002; Zhang et al., 2014). Mutations in the gene encoding SHANK3 and large duplications of the region spanning SHANK3 both cause ASD. Overexpression of SHANK3 in mice leads to SRS and maniac-like behavior (Han et al., 2013). The *Cntnap2* gene which encodes a transmembrane protein that is essential in interactions between neurons and glia is strongly associated with ASD. Deletion of *Cntnap2* leads to autistic-like behavior as well as SRS (Peñagarikano et al., 2011). 

 Along these lines, disruption of non-ion channel genes involved in many other disorders with epileptic manifestation also results in SRS in mice. Disruption of fibroblast growth factors 13 (FGF13) on the X chromosome is associated with GEFS+. Female mice in which one *Fgf13* allele was deleted exhibited SRS (Puranam et al., 2015). Leucin-rich, glioma inactivated 1 (LGI1) is a secreted protein linked to human autosomal dominant epilepsy with auditory features (ADEAF). *Lgi1* deletion in mice results in early onset SRS and seizure-related death. Selective deletion of *Lgi1* in excitatory neurons, but not parvalbumin interneurons, contributes to the epileptic phenotype associated with LGI1 (Boillot et al., 2014; Chabrol et al., 2010). The gene *Epm2a* has been indicated in an autosomal recessive disorder known as Lafora Disease. Deletion of *Epm2a* can cause spontaneous myoclonic seizures with approximately 80% penetrance at the age of 9 months (Ganesh et al., 2002). Disruption of expression of doublecortin (Nosten-Bertrand et al., 2008), synapsin (Ketzef et al., 2011), CELF4 (Yang et al., 2007) or conditional expression of a constitutively active form of MAP/ERK kinases (Nateri et al., 2007) in the murine brain all led to SRS.

Besides genetically modified mice, SRS are also found in rats and mice with *de novo* mutations reported periodically in laboratories worldwide, like GAERS rat, WAG/Rij rat, lde/lde rat and *tg*, *tg^la^*, *rkr, lh, du, stz, wgl* mice (Noebels, 2006). Among these strains, GAERS rat and WAG/Rij rat are well validated genetic models of human absence epilepsy. Spontaneous absence seizures featuring SWD first appear at P30–P40 in GAERS rat, whereas they are observed at around P60–P80 in WAG/Rij rat. SWD in both strains are fully manifested with age and last throughout their lifetime (Coenen & van Luijtelaar, 2003; De Sarro et al., 2015; Marescaux et al., 1992). The progression of absence seizures with age in WAG/Rij and GAERS rats resembles genetically-determined epileptogenesis similar to post-brain insult epileptogenesis (Russo et al., 2016).

### SRS in acquired models of epilepsy

It is estimated that up to 50% of all epilepsy cases are initiated by neurological insults also known as acquired epilepsy. To model acquired epilepsy in rodents, an episode of prolonged seizures, namely status epilepticus (SE), is commonly induced to trigger SRS (Table 2). 

**2:** SRS in acquired models of epilepsy

Insult		Methods	Features
SE	Pilocarpine (in the presence or absence of lithium)	Systemic or intracerebral injection	High mortality in general and wide spread brain damage^*^
	Kainic acid (KA)	Systemic or intracerebral injection	Hippocampal restricted damage. Short latent period (e.g., 3–5 days, KA amygdala infusion in mouse)
	Bicuculine after a lesion induced by DSP-4	Microinjection into anterior piriform cortex of rat	30% developed SRS with mossy fiber sprouting
	Tetanus toxin	Unilateral intrahippocampal injection in P10 rat	Early-life brain insult triggered diverse epileptiform response in adult rats
	Febrile seizures	Hyperthermia in P10 rat	Mimic etiology of TLE. 35.2% rats developed SRS in adults
	Sustained electrical stimulation	In BLA or AB of rat	Overall 80% (BLA) and 67% (AB) rats developed SRS
TBI		CCI or LFP	<50% developed SRS following TBI with long latent period^*^
Ischemia/hypoxia		Unilateral carotid ligation with hypoxia in P7 rat or global hypoxia in P10 rat	100% rats developed SRS, which propagated along time
Methylazoxymethanol		In utero exposure	2 out of 11 rats developed SRS
Virus infection		Intracerebral infection with Theiler’s murine encephalomyelitis virus	75% mice developed seizures 3–10 days post infection^*^
Kindling	Over electrical kindling	Repeated daily electrical stimulus for weeks and months	Labor intensive, SRS have not been well characterized
	Flurothyl kindling	Repeated flurothyl induced convulsive seizures for 8 days (once/day)	SRS were observed within the first week following flurothyl kindling then remitted^*^
^*^: model or strain dependent phenotype; SE: status epilepticus; TBI: traumatic brain injury; KA: Kainic acid; DSP-4: N-(2-Chloroethyl)-N-ethyl-2-bromobenzylamine hydrochloride; TLE: temporal lobe epilepsy; SRS: spontaneous recurrent seizures; BLA: basolateral amygdala; AB: angular bundle: CCI: controlled cortical impact; LFP: lateral fluid percussion.				

### Post-SE models

Kainic acid (KA, an ionotropic glutamate receptors agonist) and pilocarpine (a cholinergic muscarinic agonist) are two of the most commonly used chemicals to trigger SE (Ben-Ari, 1985; Ben-Ari et al., 1980; Turski et al., 1987, 1989). Systemic or intracerebral administration of KA causes SE followed by the emergence of SRS with remarkable histopathological correlation of hippocampal sclerosis in both rats and mice (Lévesque & Avoli, 2013). Compared to KA, pilocarpine-induced SE (in the presence or absence of lithium) results in higher mortality and wider spread brain damage in general along with SRS. The latency to onset of SRS and frequency of SRS varies depending on dose and administration route of chemicals as well as strains of animal. Convulsive SE can also be induced by microinjection of bicuculine into the anterior piriform cortex after a lesion of the locus coeruleus, which results in SRS in rat (Giorgi et al., 2006). In addition to chemically-induced convulsive SE, convulsive or non-convulsive SE can be induced by sustained electrical stimulation in the angular bundle or the basolateral amygdala of a rat, and can evoke SRS along with hippocampal sclerosis (Brandt et al., 2003; Gorter et al., 2001; Norwood et al., 2010). SE that occurred during early developmental stage can also cause SRS in adults. Unilateral injection of tetanus toxin into the hippocampus of P10 rats produces recurrent seizures for one week followed by epileptiform burst discharges (electrographic seizures on rare occasions) in adults (Jiang et al., 1998a; Lee et al., 1995). Both longitudinal and retrospective clinical studies reveal early life febrile SE causes temporal lobe epilepsy (TLE) in adults. Similarly, prolonged febrile seizures induced by hyperthermia in P10 rats render 35.2% of them epileptic in adulthood (Dubé et al., 2006).

### Brain insults

SRS can also develop following direct brain insults such as traumatic brain injury (TBI), stroke and viral infection in both human and rodents in the absence of SE. TBI caused by controlled cortical impact (CCI) or lateral fluid-percussion injury (FPI) is able to elicit SRS in rats and mice (Bolkvadze & Pitkänen, 2012; D'ambrosio et al., 2004; Hunt et al., 2009; Kharatishvili et al., 2006). Rats that experienced global hypoxia at P10 or hypoxic-ischemic insult at P7 developed progressive SRS in adulthood (Kadam et al., 2010; Rakhade et al., 2011; Williams et al., 2004). Rats exposed to methylazoxymethanol in utero exhibited altered GluRs expression and developed sporadic SRS in adulthood (Harrington et al., 2007). Viral encephalitis of the CNS causes severe brain damage and epilepsy. Libbey et al. described the first mouse model of viral-induced epilepsy after intracerebral infection with Theiler's murine encephalomyelitis virus, where the seizures were transient and remitted after 10 days post infection (Libbey & Fujinami, 2011; Libbey et al., 2008).

### Kindling models

Kindling is the process in which a train of repeated subconvulsive or subthreshold stimuli (electrical, audiogenic or chemical) renders a naïve animal more susceptible to subsequent stimuli. Kindling is a canonical model used for the study of epileptogenesis, yet it receives increasing criticism due to the lack of SRS. However, over-electrical kindling ultimately results in SRS (Kogure et al., 2000; McIntyre et al., 2002). Recent research revealed eight day consecutive flurothyl-kindling is sufficient to elicit SRS immediately after kindling, which remits weeks later (Kadiyala et al., 2016). 

## CONCLUDING REMARKS

Chronic rodent SRS recording is fundamental to preclinical study of epilepsy. A lack of standard methodology for SRS recording hampers the reproducibility of available models as well as the discovery of novel animal models of SRS. We recommend chronic 24/7 simultaneous video-EEG recording for rigorous study of SRS in rodents, and the recording period should vary from weeks to months depending on the model that is being used. Exclusive EEG recording often results in false positives because movement artifacts from grooming, drinking, and eating frequently generate epileptiform-like activity with rhythmic increases of frequency and amplitude (Figure 1B, right panel). Simultaneous analysis of behavior and EEG is necessary because exclusive video monitoring commonly fails to identify focal seizures or absence seizures since these lack overt behavioral manifestations.

While there are many ways to model SRS in rodents, the researcher first needs to decide what type of epilepsy they want to most closely recapitulate. Idiopathic or acquired epilepsy? TLE or absence seizures? Then the researcher needs to weigh the risks and benefits of each model that is chosen by studying the mortality and success rates and taking into consideration the developmental stage, length of latent period, frequency of SRS, electrographic and behavioral features of SRS, etc. Successful implication of rodent model of SRS will facilitate our knowledge of epilepsy and finally lead to discovery of potential biomarkers and therapies.

## ACKNOWLEDGEMENTS

We thank Kamesh Krishnamurthy (Duke University, USA) for critical discussions and reading of the manuscript.
